# Traffic-Related Air Pollution and Stress: Chen and Brauer Respond

**DOI:** 10.1289/ehp.11863R

**Published:** 2008-09

**Authors:** Edith Chen, Michael Brauer

**Affiliations:** University of British Columbia, Vancouver, British Columbia, Canada, E-mail: echen@psych.ubc.ca

We thank Clougherty and Kubzansky for their thoughtful review of our article (Chen et al. 2008). We view our article, as well as their article on exposure to violence, air pollution, and asthma etiology (Clougherty et al. 2007), as suggestive regarding how the social and physical environments operate in asthma. Although the nature of the interaction effects were different in these two studies, the broader point—that there are interactive effects between the social and physical environments in asthma —is consistent and is the key message that we wish to emphasize.

We would like to address their specific comments. First, regarding temporal issues, Clougherty and Kubzansky raise the possibility that stress increases susceptibility to subsequent pollution. We agree that this is possible; we also recognize the possibility that chronic pollution exposure could heighten responses to subsequent stressors. As we stated in our “Discussion” (Chen et al. 2008), the time frame of assessments that were available to us for these analyses was not ideal, and future studies should more specifically coordinate the timing of exposures to both stress and air pollution.

Second, we agree it is possible that saturation effects may occur at high levels of pollution exposure. However, because pollution levels in Vancouver (British Columbia, Canada) are not extreme (the range in our sample was 10–30 ppb nitrogen dioxide), we think this is an unlikely explanation.

Third, regarding spatial covariance, in our study (Chen et al. 2008), family stress was measured at the individual level; thus, we do not have neighborhood-level stress maps or information on spatial patterns in stress. Although spatial covariance between socioeconomic status and air pollution has the potential to lead to confounding, the availability of individual measures of stress and air pollution exposure estimates at the resolution of individual addresses allowed us to evaluate interactions. Our longitudinal findings also diminish the likelihood of confounding. Further, previously published pollution maps (Henderson et al. 2007) have shown that, in our study area, air pollution levels are not spatially correlated with neighborhood socioeconomic status [e.g., see UBC (University of British Columbia) Centre for Health and Environment Research 2008].

Fourth, we presented information about disease characteristics in Table 1 (Chen et al. 2008). We also controlled for asthma severity and medication use in all analyses, as described in our article under “Potential confounders.”

Finally, we agree that it would be interesting to know whether stress by air pollution effects vary by age. However, given the limited sample size in our study, we were unable to test this possibility.
